# Simulation and Experimental of Infiltration and Solidification Process for Al_2_O_3(3D)_/5083Al Interpenetrating Phase Composite for High Speed Train Prepared by Low-Pressure Infiltration

**DOI:** 10.3390/ma16206634

**Published:** 2023-10-11

**Authors:** Yanli Jiang, Pianpian Xu, Chen Zhang, Fengjun Jin, Yichao Li, Xiuling Cao, Liang Yu

**Affiliations:** 1 Key Laboratory of New Processing Technology for Nonferrous Metals & Materials, Guilin University of Technology, Guilin 541004, China; 2010043@glut.edu.cn (Y.J.); 17375063927@163.com (P.X.); zc405716298@163.com (C.Z.); jfj_6868@163.com (F.J.); y13833243036@163.com (Y.L.); 2 Hebei Technology Innovation Center for Intelligent Development and Control of Underground Built Environment, Shijiazhuang 050031, China; 3 School of Exploration Technology and Engineering, Hebei GEO University, Shijiazhuang 050031, China; 4 Collaborative Innovation Center for Exploration of Nonferrous Metal Deposits and Efficient Utilization of Resources, Guilin University of Technology, Guilin 541004, China; 5 Guangxi Modern Industry College of Innovative Development in Nonferrous Metal Material, Guilin 541004, China

**Keywords:** Al_2_O_3_3D/5083Al, numerical simulation, Infiltration, solidification, ProCAST

## Abstract

Understanding the infiltration and solidification processes of liquid 5083Al alloy into Al_2_O_3_ three-dimensional reticulated porous ceramic (Al_2_O_3(3D)_ RPC) is essential for optimizing the microstructure and properties of Al_2_O_3(3D)_/5083Al interpenetrating phase composites (IPCs) prepared by low-pressure infiltration process (LPIP). This study employs ProCAST software to simulate the infiltration and solidification processes of liquid 5083Al with pouring velocities (PV) of 0.4 m/s infiltrating into Al_2_O_3(3D)_ RPC preforms with varying porosities at different pouring temperatures (PT) to prepare Al_2_O_3(3D)_/5083Al IPCs using LPIP. The results demonstrate that pore diameter of Al_2_O_3(3D)_ RPC preforms and PT of liquid 5083Al significantly influence the of the infiltration. Solidification process analysis reveals that the Al_2_O_3(3D)_ RPC preform with smaller pore diameters allows the lower pouring velocity of 5083Al to solidify faster compared to the preform with larger pore diameters. Al_2_O_3(3D)_/5083Al IPCs were prepared successfully from Al_2_O_3(3D)_ RPC porosity of 15 PPI with liquid 5083Al at PV 0.4 m/s and PT 800 °C using LPIP, resulting in nearly fully dense composites, where both Al_2_O_3(3D)_ RPCs and 5083Al interpenetrate throughout the microstructure. The infiltration and solidification defects were reduced under air pressure of 0.3 MPa (corresponding to PV of 0.4 m/s) during LPIP. Finite volume method simulations are in good agreement with experimental data, validating the suitability of the simplified model for Al_2_O_3(3D)_ RPCs in the infiltration simulation.

## 1. Introduction

Interpenetrating Phase Composites (IPCs), often referred to as co-continuous composites, are a class of materials where both metal and ceramic phases are topologically co-continuous and three-dimensionally interconnected, forming an intricate network structure [1]. In these composites, the continuous metallic network efficiently bridges cracks, while the ceramic phase redistributes stress, facilitates load transfer, and maintains dimensional stability at elevated temperatures [2]. Metal/ceramic IPCs are known for their exceptional strength, toughness, low thermal expansion coefficient, resistance to fatigue, wear, and corrosion [3]. The fabrication of metal/ceramic IPCs typically involves creating open-porous ceramic preforms and infiltrating them with molten metal [4]. Various techniques, including replica templates, direct foaming, freeze-casting, and sacrificial pore-forming agents, have been employed to prepare ceramic preforms with specific pore geometries [5].

One of the common methods for creating metal/ceramic IPCs is the Low-Pressure Infiltration Process (LPIP), where liquid metal is injected and solidified within open-porous ceramic preforms [6,7]. However, the LPIP process is complex, involving heat transfer, fluid mechanics with phase change, and the occurrence of defects such as shrinkage and porosity [5]. Quality control in metal/ceramic IPCs prepared via LPIP depends on various factors, including the geometry of the ceramic preforms, applied pressure, pouring velocities, pouring temperatures, and the behavior of the liquid metal [8]. Understanding and predicting infiltration and solidification defects are crucial for ensuring the quality of IPCs. To gain insights into the LPIP process and improve quality control, numerical simulations have been employed to study the infiltration of open-porous ceramic preforms with metallic alloys and predict solidification defects in IPCs. Previous research has used various numerical methods, such as the volume of fluid method, porous medium model, and finite element method, to describe the flow and solidification phenomena [9,10,11,12,13,14,15,16,17]. Despite these efforts, current numerical models often simplify the preform as a single-scale porous medium and describe LPIP in simple configurations, lacking free surface tracking and comprehensive solidification modeling. Thus, the development of advanced 3D models that consider these aspects is imperative.

The 5XXX series Al-Mg alloys, known for their excellent mechanical properties, lightweight nature, corrosion resistance, and weldability, are commonly used in shipbuilding for top structures and hulls [18]. The 5083Al alloy, in particular, contains supersaturated Mg (>3.5 wt%) to enhance solid solution strengthening [19]. In previous work, Al_2_O_3_ three-dimensional reticulated porous ceramic (Al_2_O_3(3D)_ RPC) preforms were prepared and used in Al_2_O_3(3D)_/5083Al IPCs, demonstrating exceptional corrosion resistance attributed to the interface between Al_2_O_3(3D)_ RPC preforms and the 5083Al matrix [20]. Key parameters affecting the interface, including the rheology of the Al_2_O_3_ ceramic slurry, adhesion with the organic sponge replica, and cell size of the replica, were identified as critical. To optimize the interface and reduce defects in Al_2_O_3(3D)_/5083 IPCs, it is essential to thoroughly study the infiltration and solidification processes during their manufacture. Although previous research has extensively modeled metal infiltration and solidification processes, limited work has focused on the evolution of Al_2_O_3(3D)_/5083Al IPCs.

In this study, Al_2_O_3(3D)_ RPC preforms were simplified into periodic geometric shape arrays using Kelvin cells, and the infiltration and solidification processes of liquid 5083Al into Al_2_O_3(3D)_ RPC via LPIP were simulated using ProCAST software. The results were combined with experimental data to investigate factors influencing the infiltration and solidification of Al_2_O_3(3D)_/5083 IPCs, ultimately leading to process optimization.

## 2. Digital Analogue

### 2.1. Geometrical Model

**Figure 1:**
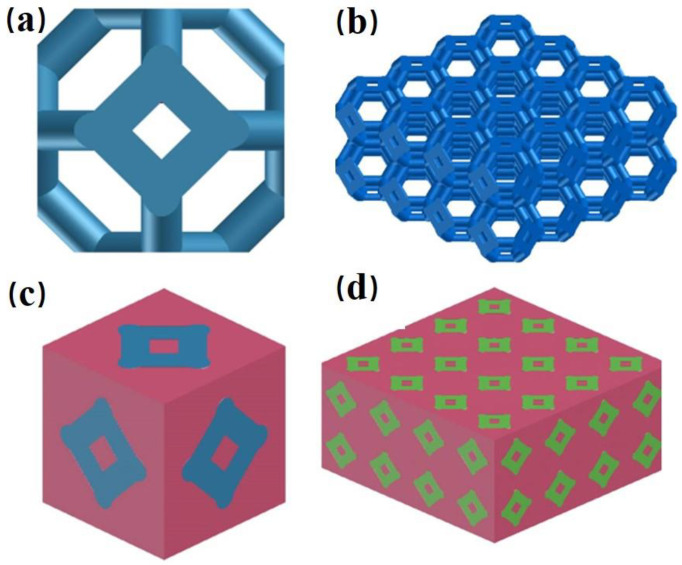
Al_2_O_3(3D)_/5083Al IPCs model. (**a**) Kelvin cell model; (**b**) Al_2_O_3(3D)_ RPC model; (**c**) infiltration unit; (**d**) Al_2_O_3(3D)_/5083Al IPCs model.

The Al_2_O_3(3D)_/5083Al IPCs three-dimensional models in Figure 1 have been generated with the software SolidWorks 2018. The Kelvin cell model was utilized to represent Al_2_O_3(3D)_ RPC preforms, as illustrated in Figure 1a. The cell length, pore size and struct diameter of the Al_2_O_3(3D)_ RPC preforms is 3 mm, 2.3 mm (approximately equivalent to 15 PPI) and 3 mm, respectively. The infiltration process involves the flow of liquid metal (5083Al) through a porous Al_2_O_3(3D)_ RPC preform. The smaller the pore size of the preform, the greater the resistance to the flow of liquid metal. As a result, there is a loss of energy of the liquid metal as it moves through the preform. This loss of energy can lead to variations in porosity within the preform, with smaller pores being filled earlier than larger ones. Higher porosities result in uneven distribution or significant variations in pore size, which can negatively affect the uniformity of penetration, so that parts of the pore area will not be well filled, resulting in reduced performance of the test sample. An increase in porosity may lead to changes in the curing behaviour within the preform, with large porosities tending to accelerate the curing behaviour, resulting in the creation of internal porosity. The infiltration domain of Al_2_O_3(3D)_ RPC model was made up with 32 Kelvin cells obtained by array processing of the infiltration cell along the x, y, z-direction, respectively [21]. Al_2_O_3(3D)_ RPC Kelvin cells represented as a network of open cells with typical 12–14 pentagonal or hexagonal faces. The infiltration unit with blue was combined with 5083Al with pink to form a single infiltration unit as depicted in Figure 1c. The Al_2_O_3(3D)_/5083 IPCs model was obtained by array processing of the infiltration unit as depicted in Figure 1d. The chemical composition of 5083Al is presented in Table 1.

**Figure 2:**
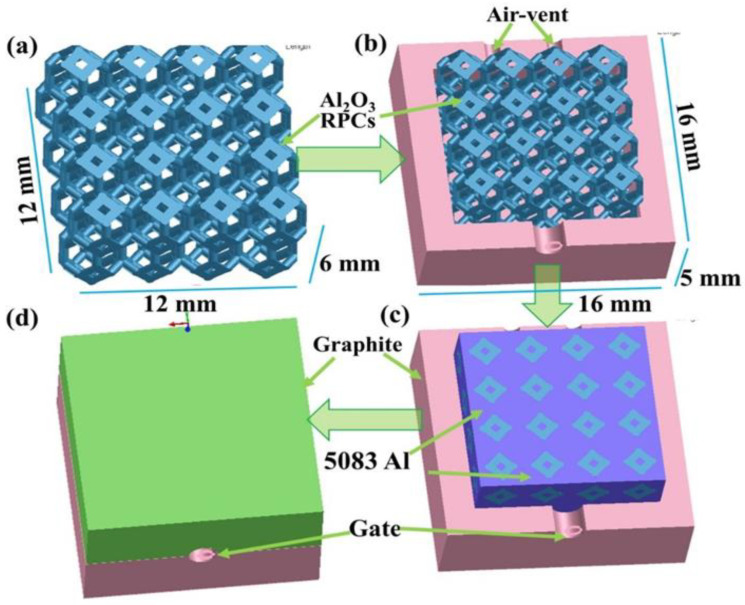
Al_2_O_3(3D)_/5083Al IPCs model for infiltration and solidification simulation during LPIP. (**a**) Al_2_O_3(3D)_ RPC of Kelvin model; (**b**) Al_2_O_3(3D)_ RPC of Kelvin model was placed in graphite upper mold; (**c**) schematic diagram of infiltration process; (**d**) Al_2_O_3(3D)_/5083Al IPCs clamping model.

A three-dimensional model generated with the software SolidWorks 2018 was used to simulate the infiltration and solidification process of Al_2_O_3(3D)_/5083Al IPCs during LPIP as depicted in Figure 2. Figure 2a shows the Al_2_O_3(3D)_ RPC of Kelvin model with dimensions of 12 × 12 × 6 mm^3^. Figure 2b displays the Al_2_O_3(3D)_ RPC of Kelvin model was placed in graphite upper mold with dimensions of 16 × 16 × 5 mm^3^. Figure 2c presents a schematic diagram of the infiltration process. The blue purple part represents 5083Al. After merging the upper and lower molds with dimensions of 16 × 16 × 10 mm^3^ was represented in Figure 2d. The clamping model in Figure 2d includes an impregnation mouth at the bottom with a diameter of 10 mm and two vents at the top with a diameter of 2 mm. 

### 2.2. Governing Equations

The transient temperature distribution and solidification velocities were calculated by finite volume method using the momentum conservation equation, mass conservation equation, and energy conservation equation expressed in the literatures [9,15,22]. In order to achieve a complete description of the infiltrating process, the flow velocities of liquid 5083Al at various positions were provided by solving the Navier–Stokes equations given in ref. [9]. The Navier–Stokes equations in matrix zone are given by

(1) $$\;\rho \frac{Dv}{Dt}=-▽\tau -▽P+\rho G$$

where *ρ* is density of molten metal (In this work assumed the liquid metal to be incompressible, which means *ρ* keeps constant); *t* is flow time; *P* is pressure at certain position; *u*, *v*, *w* is the velocity in *x*, *y*, *z* direction, *g_x_*, *g_y_*, *g_z_* is accelerated velocity in *x*, *y*, *z* direction, respectively [9].

The heat exchange between the graphite mould, Al_2_O_3(3D)_ RPC and liquid 5083Al resulted in decreasing temperature during LPIP, which changed the liquid 5083Al thermophysical parameters, such as specific heat and viscosity. The thermophysical parameters material data of 5083Al, graphite mould and Al_2_O_3(3D)_ RPC were obtained directly from the database of PROCAST software [23,24]. The governing equations given of the heat and mass transfer in REF [21] were solved using ProCAST software in this paper. Density and specific heat of Al_2_O_3_/water nanofluid are evaluated by means of the correlations proposed by Khanafer et al.

(2) $$\rho_{nf}=\varphi \rho_{np}+\left(1-\varphi \right)\rho_{bf}$$

And

(3) $$\;c_{p,nf}=\frac{{\varphi (\rho c_{p})}_{np}+(1-\varphi ){\left(\rho c_{p}\right)}_{bf}}{\rho_{nf}}$$

where the subscripts *np* and *bf* specify the nanoparticles and the base fluid, respectively, and *ϕ* indicates the nanoparticle volumetric concentration [9].

### 2.3. Numerical Procedure

**Figure 3:**
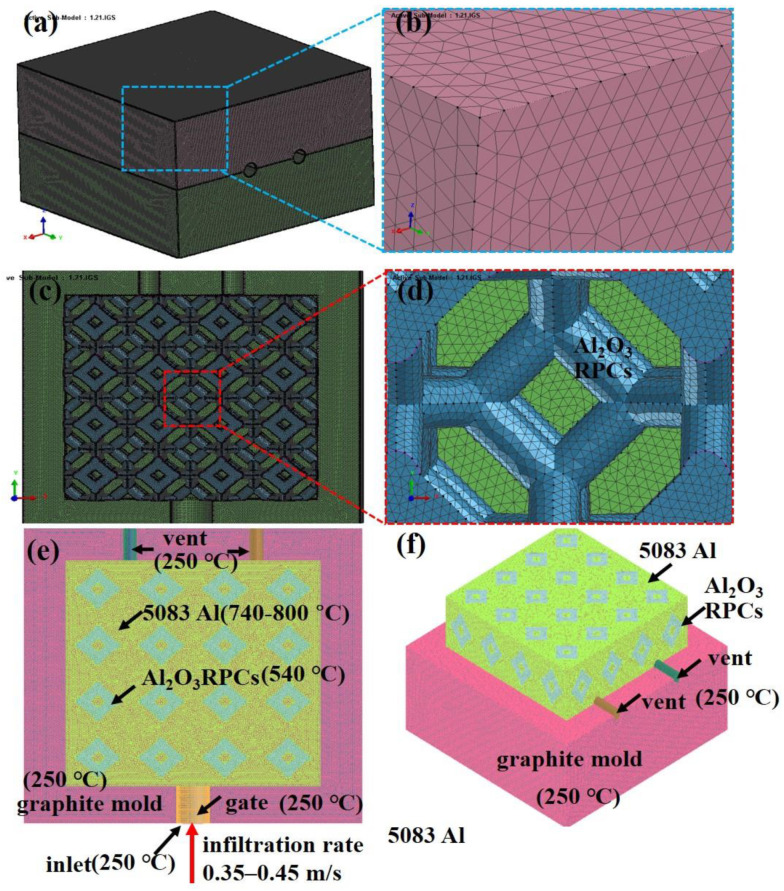
Boundary and mesh of Al_2_O_3(3D)_/5083 IPCs simulated model during LPIP. (**a**) surface mesh; (**b**) zoom of mesh; (**c**) volume mesh; (**d**) zoom of volume mesh;(**e**) front view of boundary; (**f**) side view of boundary.

Figure 3 shows the boundary and mesh of Al_2_O_3(3D)_/5083 IPCs models during LPIP. The integrity surface mesh was composed of triangles, which was divided into 160 × 160 × 100 cells, resulting in 25,600 more surface cells and 16,000 less surface cells shown in Figure 3a,b. The model was divided into approximately 180,000 volume mesh cells, which provides sufficient calculation accuracy shown in Figure 3c,d. The inlet was defined with a uniform velocity boundary condition, while all other solid surfaces were set as nonslip and nonpenetrating boundaries. As showed in Figure 3e,f, the pouring velocities (PV) of liquid 5083Al was set to 0.4 m/s, corresponding to an infiltration pressure (inlet pressure) of about 0.3 MPa, which was the pressure commonly used in low-pressure casting machines [12]. The outlet pressure is 0 Pa (absolute pressure minus atmospheric pressure is 0 Pa). The initial temperature and heat transfer coefficients (HTC) applied to each volume and boundary are listed in Table 2. Pouring temperature (PT) of liquid 5083Al was set to 740–800 °C. Initial temperature of graphite inlet, graphite gate, and graphite mold were set to 250 °C. Initial temperature of Al_2_O_3(3D)_ RPC was set to 540 °C. Liquid 5083Al was considered an ideal fluid for density calculations, and the effect of gravity was included in the momentum equation. Due to the low PT of liquid 5083Al, the radiation of liquid 5083Al into infiltrating Al_2_O_3(3D)_ RPC preform was not considered. The tip resistance can be ignored for liquid 5083Al flow in the pores is in the form of steady-state flow. Considering the liquid 5083Al as an incompressible homogeneous fluid and assuming the 5083Al flow in Al_2_O_3(3D)_ RPC preforms was a laminar flow. There was no residual air in the Al_2_O_3(3D)_ RPC preform, so the gas anti pressure was not considered. Fluid properties were defined as variables, and the momentum equation was coupled to the energy equation. The simulation employed a double precision coupling algorithm to couple the velocities. The second-order upwind advection model was used for the momentum equation, turbulent kinetic energy equation, and turbulent energy dissipation equation. The convergence criterion was set to 10^−5^. 

## 3. Material Preparation and Material Characterization

### 3.1. Material Preparation

Al_2_O_3(3D)_ RPC was prepared using replica methods in this paper. Replica methods often referred to as the lost mold process or Schwartzwalder method, have been frequently utilized to produce reticulated porous ceramics with large interconnected pores [25]. The detailed steps are as follows: (1) A three-dimensional mesh polyurethane sponge from Shenzhen Lvchuang Environmental Protection Filter Materials Co., Ltd. (Shenzhen, China) was immersed in a NaOH solution for 18 h to remove the interlayer film and increase surface roughness. The purpose is to improve the adhesion between the polyurethane sponge surface and the Al_2_O_3_ slurry. (2) The sponges used as templates were cut into a circle with a diameter of 100 mm and a thickness of 8 mm. (3) The sponge was impregnated into Al_2_O_3_ slurry. The impregnated sponge body was then passed through rollers to drain the surplus slurry and maintain the ceramic content in the infiltrated body. (4) The ceramic-coated template was subsequently dried in a microwave oven for 15 min to obtain a green alumina mesh porous body with a well-defined structure. (5) The pyrolyzed through careful heating to 400 °C for 2 h decomposed or burned out the polyurethane sponge templates. (6) In a graphite resistance furnace from Jinzhou Santai Electric Furnace Factory, China, with argon gas as the sintering atmosphere at 1600 °C for 3 h, the ceramic layers were sintered to obtain Al_2_O_3(3D)_ RPC with the same morphology as that of the original cellular polyurethane sponge template, which was approximately 15 PPI (pores per inch). 

**Figure 4:**
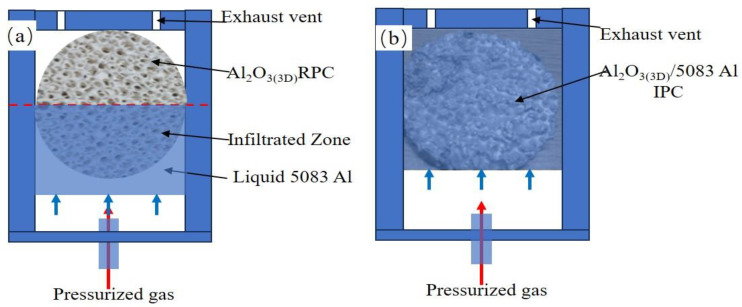
Schematic diagram of liquid 5083Al infiltrating into Al_2_O_3(3D)_ RPC using LPIP. (**a**) low-pressure infiltration process, (**b**) solidification process.

Figure 4 shows the schematic diagram of liquid 5083Al infiltrating into Al_2_O_3(3D)_ RPC using LPIP. The 5083Al was in the form of nuggets and placed in the graphite crucibles and heated from 25 °C to 800 °C for 2 h in the crucible furnace from Zhengzhou Xinhan Instrument Equipment Co., Ltd. (Zhengzhou, China). The liquid 5083Al in the graphite crucible was regularly stirred to ensure a uniform composition. Al_2_O_3(3D)_ RPC were heated to 540 °C. The Al_2_O_3(3D)_ RPC was placed on the liquid 5083Al, and pressurized gas was applied for about 20 min, as shown in Figure 4a. The liquid 5083Al completely infiltrated the Al_2_O_3(3D)_ RPC and cooled to obtain Al_2_O_3(3D)_)/5083 IPCs in Figure 4b. The simulation results obtained from ProCAST were compared and verified.

### 3.2. Material Characterization

The obtained samples of Al_2_O_3(3D)_/5083 IPCs were subjected to X-ray diffraction (XRD) analysis using Cu Kα radiation at 40 kV and 100 mA, employing a computer-controlled diffractometer (PANALYTICL B.V/PW3040/60, Netherlands). The XRD data were recorded in continuous scanning mode with a scanning angle (2θ) ranging from 10° to 90° and a scanning rate of 0.02°/s. The microstructure of the samples of Al_2_O_3(3D)_/5083 was characterized using scanning electron microscopy (SEM) at 15 kV and 10 mA. The composition of the material was analyzed using energy-dispersive spectroscopy (EDS).

## 4. Results and Discussion

### 4.1. Effect of Pouring Temperature on Infiltration Depth

**Figure 5:**
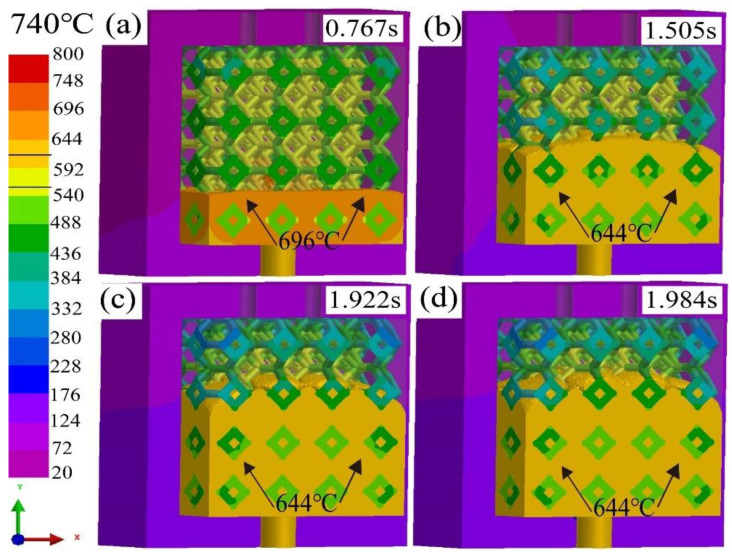
Infiltration depth of liquid 5083Al with PV of 0.4 m/s and PT of 740 °C infiltrating into Al_2_O_3(3D)_ with different infiltration times using LPIP. (**a**) 0.767 s; (**b**) 1.505 s; (**c**) 1.922s; (**d**) 1.984 s.

Figure 5 shows the simulated results of infiltration depth of liquid 5083Al with pouring velocities (PV) of 0.4 m/s infiltrating and pouring temperature (PT) of 740 °C into Al_2_O_3(3D)_ RPC with different times in infiltrating stage using LPIP. During the initial infiltrating stage, the liquid 5083Al flowed freely upward along the vertical inlet under the influence of pressurized gas. At 0.767 s, the mold was filled to about 25%, the temperature of liquid 5083Al was 696 °C (Figure 5a). At 1.505 s, the mold was filled to about 50%, temperature of liquid 5083Al was higher than 644 °C (Figure 5b). The black arrow indicated the position where the 644 °C isotherm was located showed in Figure 5b–d. Temperature of liquid 5083Al was still above its solidus temperature, and the infiltrating process could continue. However, at 1.922 s, the mold was filled to about 70%, temperature of the liquid 5083Al was below 644 °C (Figure 5c). Temperature of liquid 5083Al was lower than the solidus temperature, and liquid 5083Al began to solidify. The mold infiltrating could not continue. The final infiltration depth was defined as the maximum length of Al_2_O_3(3D)_ preform which liquid 5083Al can percolate before the channel was completely blocked by liquid 5083Al solidification. It was evident that overall fill time was approximately 1.984 s, full impregnation was not achieved at 740 °C as well as the final infiltration depth was about 70% (Figure 5d).

**Figure 6:**
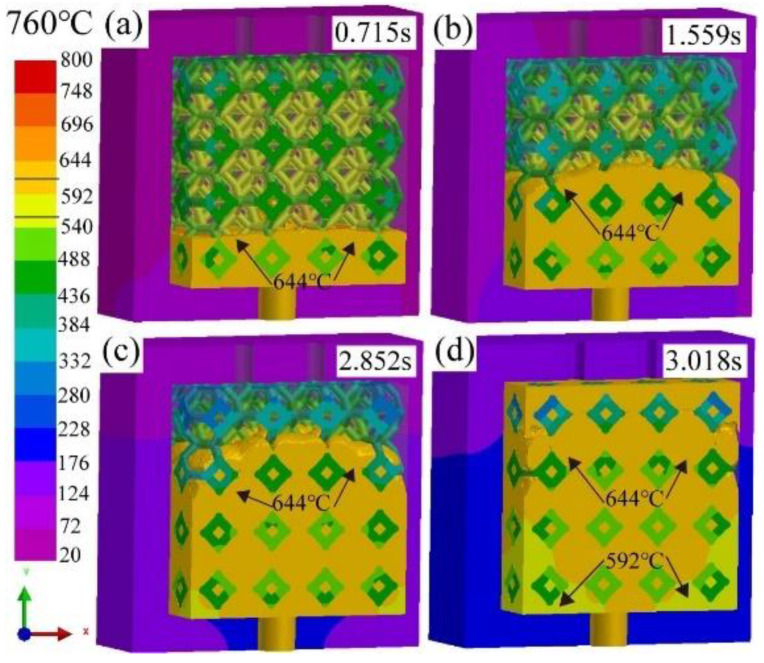
Infiltration depth of liquid 5083Al with PV of 0.4 m/s and PT of 760 °C infiltrating into Al_2_O_3(3D)_ with different infiltration times using LPIP. (**a**) 0.715 s; (**b**) 1.559 s; (**c**) 2.852 ;(**d**) 3.018 s.

Figure 6 shows the simulated results of infiltration depth of liquid 5083Al with PV of 0.4 m/s and PT of 760 °C infiltrating into Al_2_O_3(3D)_ with different times in infiltrating stage using LPIP. The black arrow indicated the position where the 644 °C isotherm was located. Compared with infiltration depth of 644 °C isotherm of liquid 5083Al with filling 20%, 50%, 70% indicated by black arrow in Figure 5, the infiltration depth of 644 °C isotherm of liquid 5083Al in Figure 6 in infiltration direction was increased by about 10%, 20%, and 30% with filling 20%, 50%, 70%, respectively. At 0.715 s, the mold was filled to about 25% (Figure 6a). At 1.559 s, the mold was filled to about 50% (Figure 6b). However, at 2.852 s, the mold was filled to about 70% (Figure 6c). The overall fill time was about 3.018 s, the final infiltration depth was about 100%, complete impregnation was achieved (Figure 6d). The microporosities of infiltration gaps at the interface between 5083Al and Al_2_O_3(3D)_ RPC or the segregation of the 5083Al matrix were observed during infiltration.

**Figure 7:**
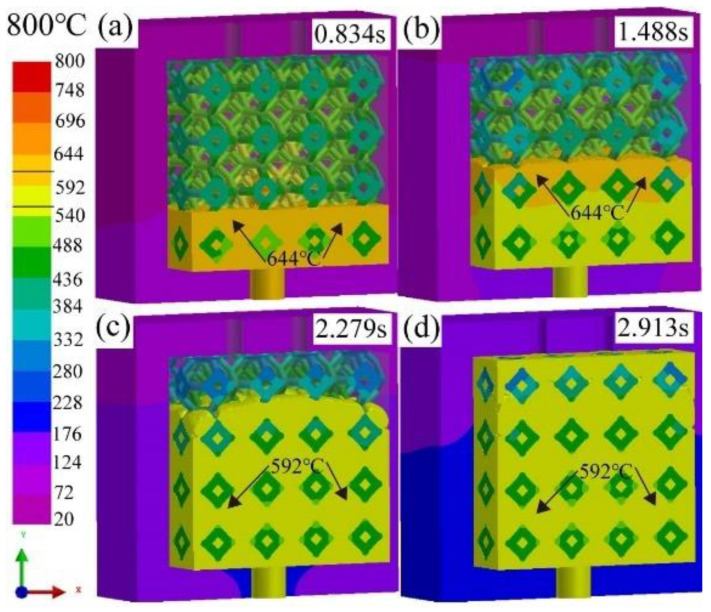
Infiltration depth of liquid 5083Al with PV of 0.4 m/s and PT of 800 °C infiltrating into Al_2_O_3(3D)_ with different infiltration times using LPIP. (**a**) 0.834 s; (**b**) 1.488 s; (**c**) 2.279 s; (**d**) 2.913 s.

Figure 7 shows the simulated results of infiltration depth of liquid 5083Al with PV of 0.4 m/s and PT of 800 °C infiltrating into Al_2_O_3(3D)_ with different times in infiltrating stage using LPIP. Compared with the depth of the 644 °C and 592 °C isotherms of liquid 5083Al with filling 20%, 50%, 70% indicated by black arrow in Figure 6, the depth of the 644 °C and 592 °C isotherms of liquid 5083Al in Figure 7 in the infiltration direction was increased by about 5%, 10%, and 12% with filling 20%, 50%, 70%, respectively. At 0.834 s, the mold was filled to about 25% (Figure 7a). At 1.488 s, the mold was filled to about 50% (Figure 7b). However, at 2.279 s, the mold was filled to about 70% (Figure 7c). The overall fill time was about 2.913 s and the final infiltration depth was about 100% (Figure 6d). Comparing the infiltration effects at these temperatures 740 °C and 760 °C, no obvious defects, and full impregnation was obtained at 800 °C. It can be observed that, the lower the PV, the more significant solidification and the lower the final infiltration depth. Increasing PT to 800 °C, predicting results showed that the interfaces of Al_2_O_3(3D)_ RPC–liquid 5083Al, and liquid 5083Al–mold experience higher temperature gradients. The viscosity of liquid 5083 decreased, result in higher infiltration velocities and shorter fill completion time.

### 4.2. Flow Field and Temperature Field of Liquid 5083Al at PT 800 °C

**Figure 8:**
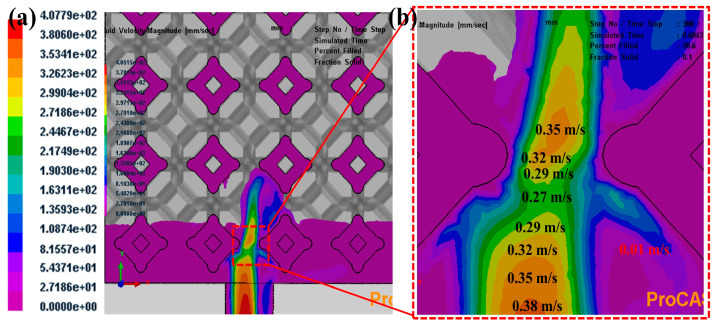
infiltration velocities along the flow direction of liquid 5083Al with PV of 0.4 m/s and PT of 800 °C infiltrating into Al_2_O_3(3D)_ using LPIP. (**a**) Overhead view; (**b**) zoom.

Figure 8 illustrates the infiltration velocities along the flow direction of liquid 5083Al with PV of 0.4 m/s and PT of 800 °C infiltrating into Al_2_O_3(3D)_ using LPIP. Liquid 5083Al was Infiltrated continuously through the bottom face of the channel at constant PV of 0.4 m/s and at constant PT of 800 °C. Because the placement of Al_2_O_3(3D)_ preform was not close to the wall of mold, the infiltration process was actually a three-dimensional multi-directional infiltration. Due to the viscous loss caused by the porous medium, the flow front became very flat. The infiltration process was relatively stable with small fluctuation, and Al_2_O_3(3D)_ preform was infiltrated completely in a very short time. During the infiltration process, the smaller the pore size of Al_2_O_3(3D)_ preform would cause the more work of resistance, the more loss of the energy of liquid 5083Al and the smaller PV. This correlation favors filling of larger pore prior to the smaller pores when the Al_2_O_3(3D)_ and liquid 5083Al system was poorly wetting. The PV decreased to 0.27 m/s at the place with the smallest pore size of the Al_2_O_3(3D)_ preform. Before liquid 5083Al reached the Al_2_O_3(3D)_ preforms, the flow front had tiny fluctuations and was not flat. This kind of flow can easily cause gas entrapment and casting defects. The reason for this is that the layered transition in temperature indicates that different regions of the material are solidifying at different rates. During solidification, as the material transitions from a liquid to a solid state, temperature gradients can develop within the material. Regions that solidify earlier will have lower temperatures, while those that solidify later will remain at higher temperatures. These temperature differences can lead to variations in the rate of solidification. The layered transition in infiltration time suggests that certain regions within the material are experiencing slower solidification rates. In some cases, this can result from a slower advancement of the solidification front in specific areas. Slower solidification rates can lead to incomplete filling of voids, creating porosity and gaps in the material. Non-uniform solidification can also contribute to the formation of shrinkage defects. As different regions solidify at different times and rates, they will undergo volume changes associated with the phase transition from liquid to solid. This non-simultaneous volume change can create internal stresses and voids, leading to shrinkage defects.

**Figure 9:**
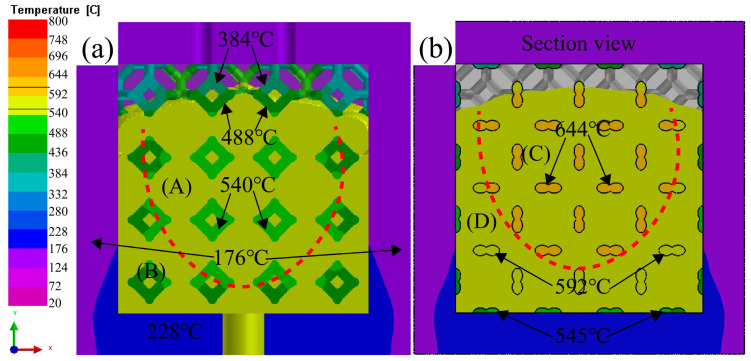
The temperature flow direction of liquid 5083Al with PV of 0.4 m/s and PT of 800 °C infiltrating into Al_2_O_3(3D)_ using LPIP. (**a**) overhead view; (**b**) section view.

Figure 9 presents the temperature along the flow direction. The results indicate PV and PT played a crucial role in determining the velocity of liquid 5083Al through the clearance and the degree of pore shrinkage at the end of infiltration. Temperature of the liquid 5083Al decreased along the flow direction in Figure 9a. The section view in Figure 9b shows the temperature in the middle was higher, while the temperature around the Al_2_O_3(3D)_ dropped. This temperature distribution may affect the different solidification rates between the middle and the surrounding parts of the casting, resulting in defects in the middle of the casting. The viscosity and flow velocity of the liquid 5083Al undergo significant changes when there is a large temperature gradient in the region. 

### 4.3. Effect of Porosity of Al_2_O_3(3D)_ on Liquid 5083Al with PV 0.4 m/s and PT 800 °C in LPIP

**Figure 10:**
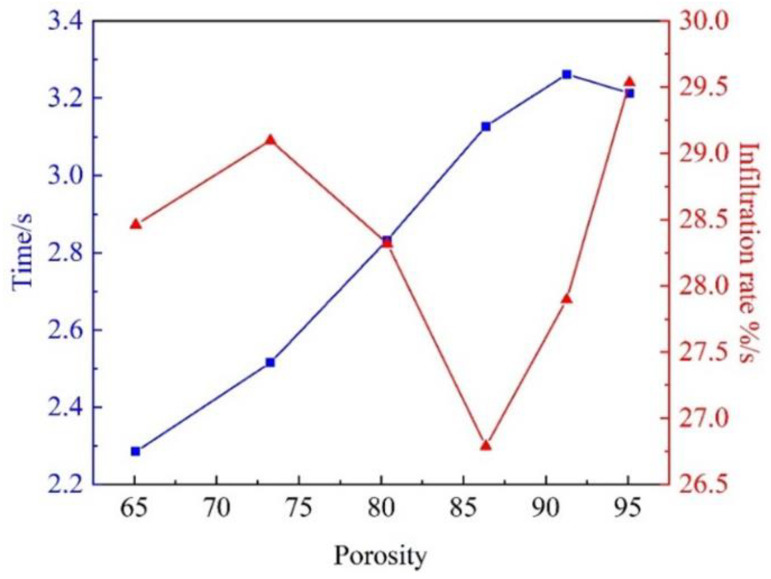
Effect of Al_2_O_3(3D)_ porosity on infiltration time and infiltration rate of liquid 5083Al with PV of 0.4 m/s and PT of 800 °C during LPIP.

The mesh numbers for 1, 2, 3, and 4 times Al_2_O_3(3D)_ RPC impregnating body were divided into 3,836,942, 3,514,000, 3,407,296, and 3,442,287, respectively. The impregnation time for 1, 2, 3, and 4 times was 3.00 s, 2.84 s, 2.94 s, and 3.490 s show in Figure 10. In most tests, the penetration rate of the whole cavity can be completed at around 0.4 m/s. The infiltration rate is calculated using Equation (1)

V = F/T(4)

where V is the infiltration rate, F is the infiltration percentage, and T is the infiltration time. When the porosity is greater than 80%, the volume rate changes at 86.4%, 91.3%, and 95.1%, and the infiltration time is 3.13 s, 3.26 s, and 3.21 s, respectively, with infiltration rates of 26.8 %/s, 27.9 %/s, and 29.5 %/s. The optimal porosity exists in the range of 65% to 80%, with the volume rate changes at 65.1%, 73.3%, and 80.4%, the infiltration time 2.28 s, 2.51 s, and 2.83 s, and the infiltration rate 28.5 %/s, 29.1 %/s, and 28.3 %/s at an infiltration rate of 0.4 m/s. Due to the large pores of Al_2_O_3(3D)_ RPC with 5 PPI, infiltration becomes easier and the flowable area of the pores increases. As the porosity increased, the flowable area of the pores becomes wider, and the pore space is quickly occupied. Thus, the fluid volume increased within the porous diameter increased. It gradually loses its guiding effect on liquid 5083Al, leading to turbulent phenomena in Figure 10. Al_2_O_3(3D)_ RPC with positively influences infiltration, improving the infiltration effect [15]. Using porosity of 65~80% improved the infiltration effect and better prepare Al_2_O_3(3D)_/5083Al.

**Figure 11:**
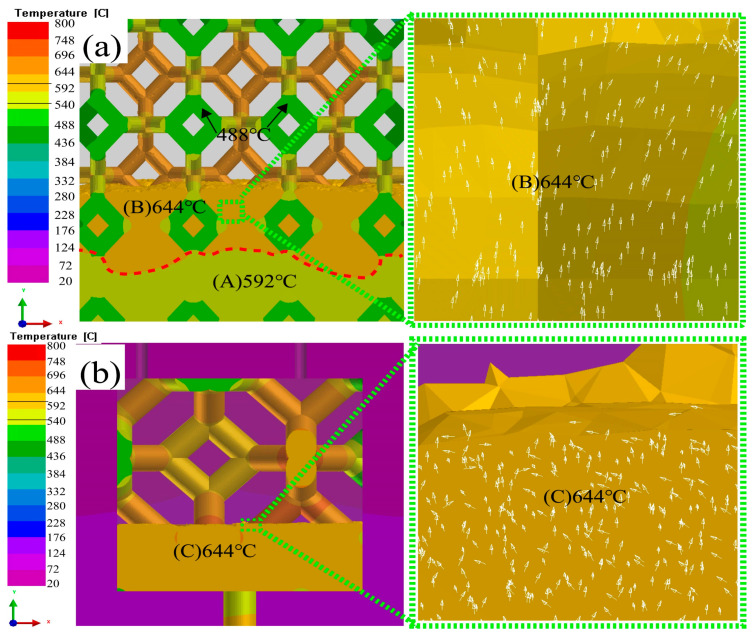
Effect of Al_2_O_3(3D)_ porosity on liquid 5083Al with PV of 0.4 m/s and PT of 800 °C during LPIP; (**a**) 15 PPI; (**b**) 5 PPI.

Figure 11 of Al_2_O_3(3D)_ with 15 PPI and 5 PPI and infiltration. Al_2_O_3(3D)_ RPC porosity closely related to the infiltration rate. at PT 800 °C, laminar flow and turbulent flow were observed in Figure 11a,b, respectively. Al_2_O_3(3D)_ with 15 PPI in in Figure 11a could guide the infiltration, which is conducive to Al_2_O_3(3D)_/5083 infiltration forming and reducing infiltration defects [26]. The infiltration decreases first and then increases using Al_2_O_3(3D)_ RPC with 5 PPI in Figure 11b.

**Figure 12:**
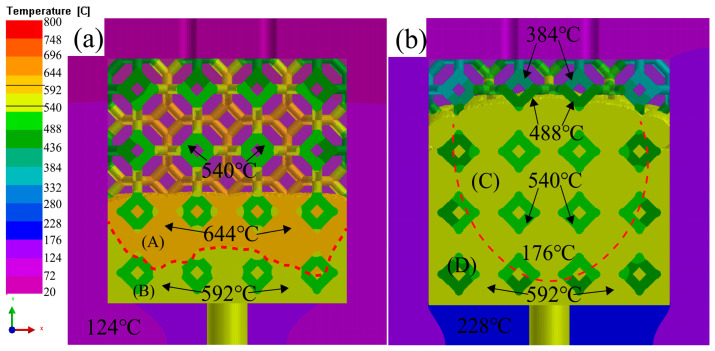
Temperature changes of graphite model with liquid 5083Al at PV of 0.4 m/s and PT of 800 °C during LPIP; (**a**) zones A, B; (**b**) zones C, D.

Figure 12 shows the temperature changes of graphite model with liquid 5083Al at PV of 0.4 m/s and PT of 800 °C during LPIP. The temperature change of Al_2_O_3(3D)_ RPC was slower than that of the graphite mold. The thermal conductivity of the graphite mold was better than that of the Al_2_O_3(3D)_ RPC, resulting in the Al_2_O_3(3D)_ RPC having a thermal insulation effect on liquid 5083Al compared to the graphite mold. The liquid 5083Al was divided into zones A, B, C, and D. The liquid 5083Al temperature in zone A was 644 °C, in zone B was 592 °C as shown in Figure 12a, and in zone C was 592 °C compared to zone D as shown in Figure 12b. The liquid 5083Al in zone A had high temperature, low viscosity, and fast infiltration rate, while the liquid 5083Al in zones B, C, and D had lower temperature, higher viscosity, and lower infiltration rate. The velocity field exhibited large fluctuations, leading to turbulence and low porosity. It is expected the results with PV of 0.4 m/s and PT of 800 °C during LPIP would help to improve the quality of combination of interfaces of Al_2_O_3(3D)_ and the 5083Al matrix.

### 4.4. Solidification Process 

**Figure 13:**
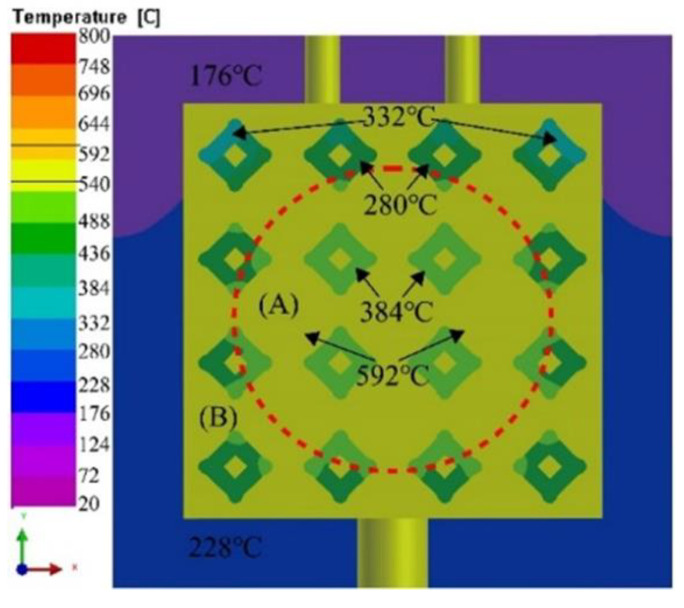
mold temperature fields during solidification process with liquid 5083Al at PV of 0.4 m/s and PT of 800 °C.

Figure 13 shows the simulation result of mold temperature fields during solidification process with liquid 5083Al at PV of 0.4 m/s and PT of 800 °C. When infiltration was completed, the temperature of the whole mold dropped. The casting was divided into zones A and B according to the temperature zone. At the completion of infiltration, the temperature of Al_2_O_3(3D)_ RPC in zone A was 384 °C, and in zone B was 332 °C. The maximum temperature of the mold surface was 228 °C. The inner temperature of the casting was higher than that of the casting.

**Figure 14:**
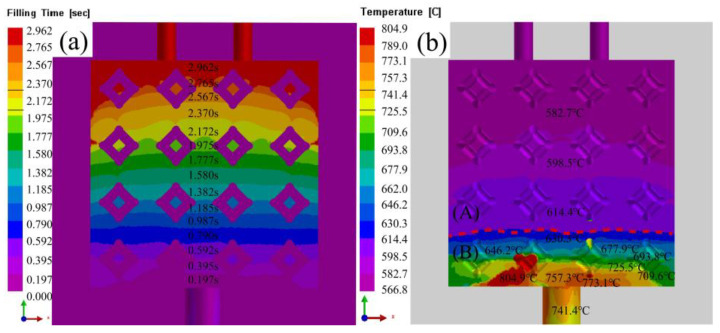
Simulation changes of time and temperature after infiltration completion with liquid 5083Al at PV of 0.4 m/s and PT of 800 °C; (**a**) time; (**b**) temperature.

Figure 14 shows the simulation changes of infiltration time and temperature after infiltration completion with liquid 5083Al at PV of 0.4 m/s and PT of 800 °C. Both the infiltration time and the infiltration temperature presented a layered transition as shown in Figure 14a,b. The infiltration time could be divided into 15 layers in Figure 14a. The first five layers of infiltration time were short, corresponding to the 0 s~0.98 s stage of stable infiltration. The middle five layers from 0.98 s to 1.97 s showed a certain upward bulge in the two layers near the top, indicating that the liquid 5083Al flow velocity slowed down in this region. At 1.79~2.96 s, in regions with significant temperature gradients, the viscosity may increase, leading to slower infiltration rates and deformations in the flow front. the overall infiltration time bar has a large deformation and bulges upward, and the infiltration time bar thickens, indicating slower infiltration at this time. The infiltration rate of liquid 5083Al decreased under the influence of gravity, making shrinkage and loosening phenomena more likely to occur. Infiltration temperature divided into three layers as shown in Figure 14b. The temperature at the bottom where 5083Al was impregnated dropped rapidly and was close to the preset temperature of Al_2_O_3(3D)_ RPC. The middle layer maintained a stable temperature between 614 °C and 598 °C, indicating stable 5083Al infiltration. The top layer had a temperature ranging from about 566 °C to 582 °C, closed to the solidification temperature of liquid 5083Al. At this stage, liquid 5083Al became sticky, and the infiltration rate decreases rapidly. The velocities of liquid 5083Al at the bottom could not meet the stable infiltration at the top, resulting in faster infiltration time in the middle than on both sides.

**Figure 15:**
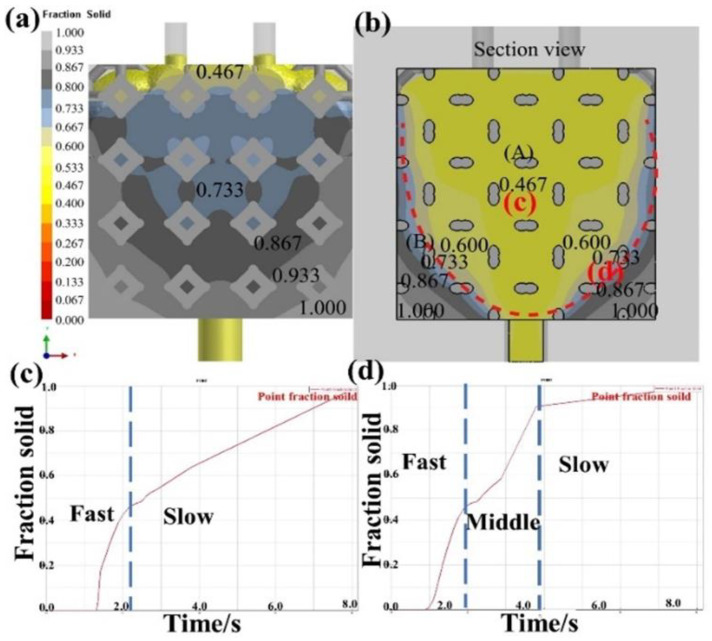
The solidification velocities of different parts and the solidification curve with liquid 5083Al at PV of 0.4 m/s and PT of 800 °C; (**a**) overhead view of fraction solid; (**b**) section view of fraction solid; (**c**) Solidification temperature curves of marked points c; (**d**) Solidification temperature curves of marked points d.

Figure 15 shows the solidification velocities of different parts and the solidification curve with liquid 5083Al at PV of 0.4 m/s and PT of 800 °C. The iteration step size was 1100, and the solidification state was centered towards the periphery. During infiltration, liquid Al flows from the bottom center to the periphery [27]. As liquid 5083Al infiltrated upward, the flow rate of liquid 5083Al slowed down. Solidification rate of the Al liquid near the inner wall of the model with heat conduction of the graphite model was faster than that of the Al liquid under Al_2_O_3(3D)_ RPC insulation. This resulted in funnel-shaped solidification of liquid 5083Al. Figure 15c,d show the solidification temperature curves of marked points (c) and (d) in Figure 15b, respectively [28]. The solidification temperature curve in Figure 15c shows two changes in velocities, and the driving force of solidification was temperature change. As the solidification developed from the inner wall of the model to the center of the casting, the solidification in Figure 15c was controlled by the heat transfer of Al_2_O_3(3D)_ RPC, resulting in a faster solidification rate [29]. When Al_2_O_3(3D)_ RPC temperature was consistent with the temperature of the aluminized liquid, the solidification changed to be controlled by the air cooling of the outer mold [2]. Figure 15d shows in the first stage, when the liquid Al contacted the Al_2_O_3(3D)_ RPC, it was controlled by the heat transfer of the Al_2_O_3(3D)_ RPC, resulting in a faster solidification rate. In the second stage, because the temperature of Al_2_O_3(3D)_ RPC was not consistent with that of liquid 5083Al, solidification was controlled by the air cooling of Al_2_O_3(3D)_ RPC and outer mold. In the third stage, the temperature of Al_2_O_3(3D)_ RPC was the same as that of liquid 5083Al, and the solidification changed to be controlled by the air cooling of the graphite mold [30].

**Figure 16:**
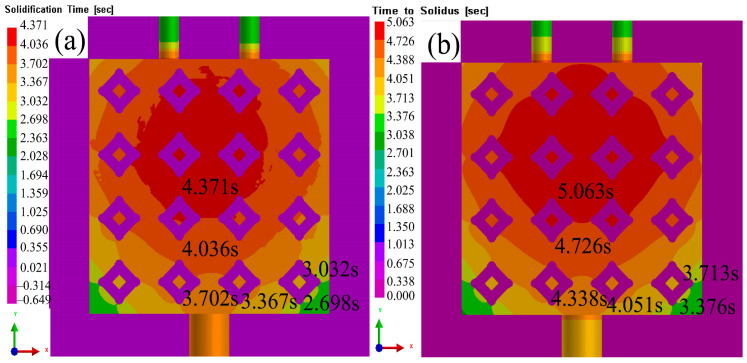
Solidification completion time and solid-phase transition completed time with liquid 5083Al at PV of 0.4 m/s and PT of 800 °C; (**a**) solidification completion time; (**b**) solid-phase transition completed time.

Figure 16a,b present a comparison of the solidification time and solid-phase transition completed time, revealing that the solidification time in the center was longer than that around it. The overall solidification process was influenced by Al_2_O_3(3D)_ RPC, resulting in a concentration of solidification time and solid-liquid phase in the center, forming a spherical diffusion pattern. The solid-liquid phase could be divided into three distinct parts [31]. The central part of the solidification processed and the time taken for the liquid phase to solidify were relatively long, indicating that the velocities of liquid 5083Al in this region was insufficient, and there was a probability of incomplete solidification leading to porosity. In contrast, the solidification time was more uniform in the peripheral regions due to the influence of the input of liquid Al and Al_2_O_3(3D)_ RPC. As a result, the time range for solid-liquid phase transition was larger than the solidification time range. Specifically, the second layer experienced a solidification time ranging from 3.7 s to 4.0 s, and the transition time from solid-liquid phase to solid was from 4.4 s to 4.7 s for the entire solidification process, which aligned with the characteristics of this part. The third layer was mainly affected by the inner wall of the model, and the infiltration rate had little impact. At about 5.0 s, solid phase transition completed Additionally, Al_2_O_3(3D)_ RPC resulted in a shorter solidification time, and the transition from solid-liquid phase to solid occurs earlier in this region [16].

**Figure 17:**
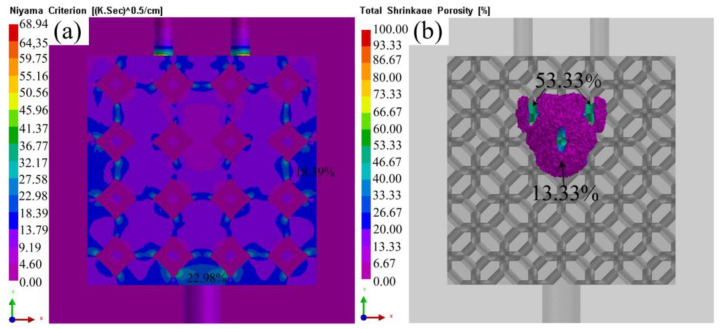
Porosity prediction and shrinkage prediction with liquid 5083Al at PV of 0.4 m/s and PT of 800 °C; (**a**) porosity prediction; (**b**) shrinkage prediction.

Figure 17 shows simulated prediction of porosity and shrinkage with liquid 5083Al at PV of 0.4 m/s and PT of 800 °C. Shrinkage pore distribution was more uniform, and the probability of shrinkage pore occurrence was small. The shrinkage porosity distribution is low and concentrated in Al_2_O_3(3D)_RPC center [17]. There was a probability of shrinkage at interface of Al_2_O_3(3D)_ RPC and 5083Al matrix, as well as certain probability of shrinkage in 5083Al matrix. The largest probability of shrinkage was the inlet part of liquid 5083Al. Due to the influence of many factors, such as residual stress concentration, the solidification temperature of liquid 5083Al in Al_2_O_3(3D)_ RPC, the increase of viscosity of liquid Al_2_O_3(3D)_ RPC, the shrinkage percentage was 13.33%, and the probability of shrinkage is small [1].

### 4.5. IPC Casting Process

**Figure 18:**
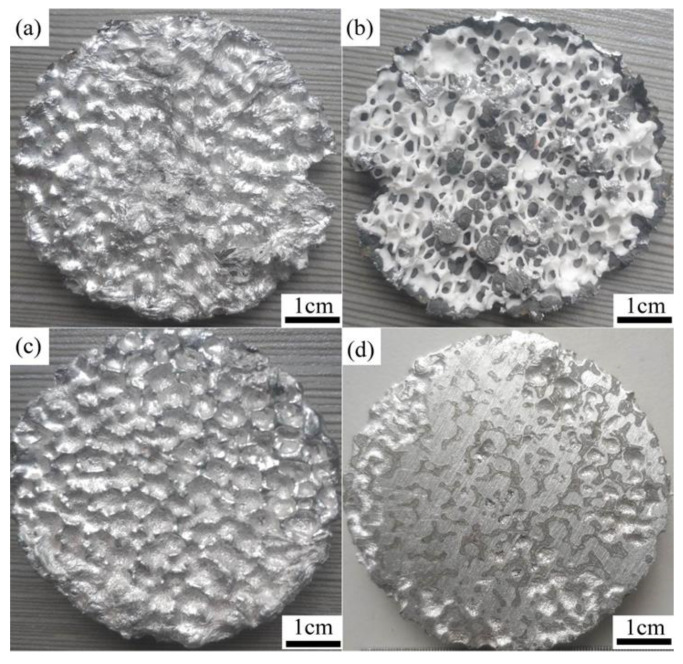
Al_2_O_3(3D)_/5083Al IPCs prepared by LPIP; (**a**,**b**) 740 °C; (**c**,**d**) 800 °C.

Liquid 5083Al was infiltrated into the as-prepared Al_2_O_3(3D)_ RPC preforms with high uniform open porosity (58–74%), pore size (3.5 mm) to fabricate Al_2_O_3(3D)_/5083Al IPCs by LPIP. For successful melt infiltration to prepare Al_2_O_3(3D)_/5083Al IPCs, the Al_2_O_3(3D)_ RPC preforms must be predominantly open porous and sufficiently strong struts without cracks or other defects. It was observed that the infiltration of the liquid 5083Al at PV of 0.4 m/s and PT of 740 °C was incomplete, and a significant amount of liquid 5083Al remained trapped inside the Al_2_O_3(3D)_ RPC. This was likely due to the excellent heat dissipation ability of Al_2_O_3(3D)_ RPC, as depicted in Figure 18a, which resulted in rapid cooling of the liquid inside Al_2_O_3(3D)_ RPC. Consequently, the infiltration inlet was obstructed by the cooled 5083Al, preventing further infiltration, as shown in Figure 18b. To address this issue, Al_2_O_3(3D)_/5083 IPCs were prepared by LPIP with liquid 5083Al at PV of 0.4 m/s and PT of 800 °C, and the infiltration process was repeated. Test sample was successfully obtained in Figure 18c. The test sample exhibited certain characteristics, such as a considerable weight, a reflective silver luster, and a solid sound without any hollow sensation upon gentle tapping. After the successful infiltration, the obtained sample, Al_2_O_3(3D)_/5083Al, was further polished, as shown in Figure 18d. The surface of the polished sample exhibited distinct features: the gray parts corresponded to Al_2_O_3(3D)_ RPC, while the metal luster indicated 5083Al. 

**Figure 19:**
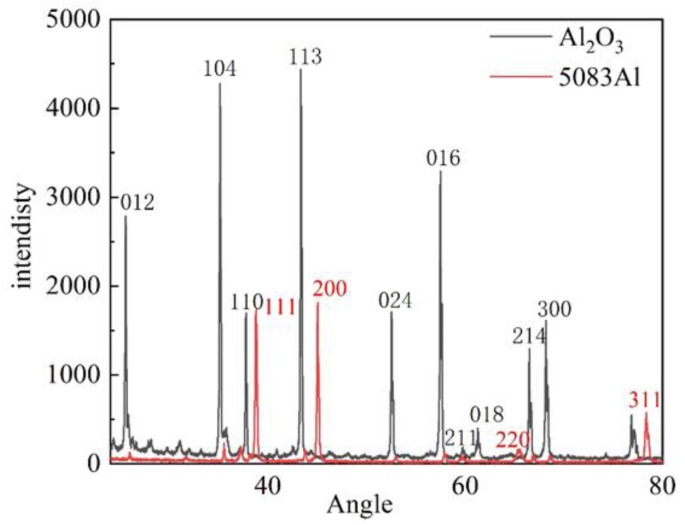
XRD patterns of Al_2_O_3(3D)_/5083Al.

Figure 19 X-ray Diffraction (XRD) testing was conducted on Al_2_O_3(3D)_/5083Al IPCs. The results were compared with standard reference cards. The XRD analysis confirmed that the Al_2_O_3(3D)_/5083Al IPCs was composed of Al_2_O_3_ and Al alloy. 

**Figure 20:**
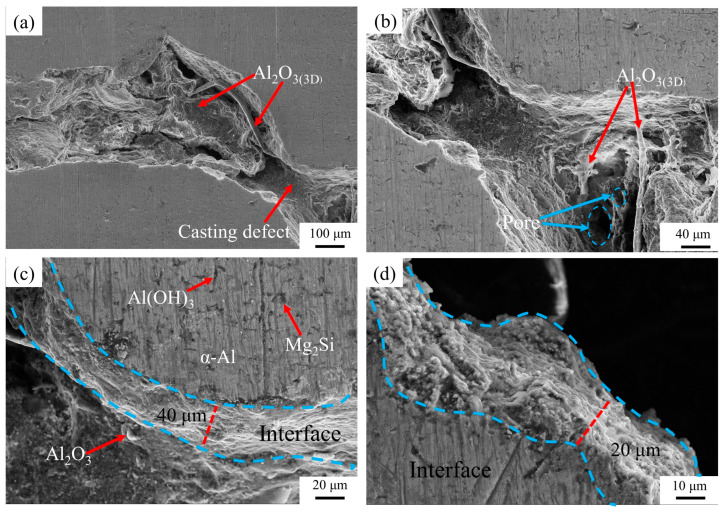
SEM images of Al_2_O_3(3D)_/5083Al from Al_2_O_3(3D)_ porosity of 15 PPI with liquid 5083Al at PV 0.4 m/s and PT 800 °C; (**a**) SEM of Al_2_O_3(3D)_/5083Al; (**b**) zoom of Al_2_O_3(3D)_/5083Al; (**c**) interface; (**d**) zoom of interface.

Figure 20 shows the SEM of surface morphology of Al_2_O_3(3D)_/5083Al. The dark color was Al_2_O_3(3D)_ RPC, which contains fine pores. The Al_2_O_3(3D)_/5083 appeared to be well bonded with no large pore defects, and the interface between the two phases was closely bonded [13]. The infiltration and solidification defects were reduced under air pressure of 0.3 MPa (corresponding to an inlet pressure of about 0.3 MPa or PV of 0.4 m/s) during LPIP. In addition, the Al_2_O_3(3D)_ RPC exhibited excellent affinity and good wettability with the liquid 5083Al under pressure, fine air bubbles were effectively minimized at the interface between the two materials until solidification crystallization completed. As the result, the interface between Al_2_O_3(3D)_ RPC and 5083Al demonstrated a strong bonding. This reduction in air bubbles helped to eliminate voids, leading to a more homogenous and structurally sound Al_2_O_3(3D)_/5083Al composite [13]. This property made it suitable for low-pressure casting applications.

**Figure 21:**
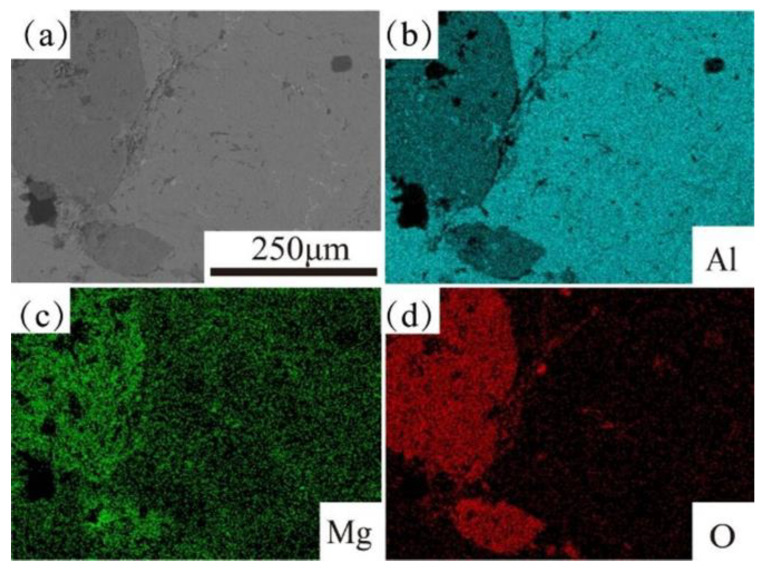
EDS map scanning of Al_2_O_3(3D)_/5083Al from Al_2_O_3(3D)_ porosity of 15 PPI with liquid 5083Al with PV 0.4 m/s at PT 800 °C. (**a**) SEM of Al_2_O_3(3D)_/5083Al; (**b**) Al; (**c**) Mg; (**d**) O.

Figure 21 shows EDS map scanning shows the element distribution in Al_2_O_3(3D)_/5083Al. Al element was clearly divided at the phase interface [31]. Mg element was enriched in Al_2_O_3(3D)_ RPC compared to 5083Al, indicating Mg diffusion towards Al_2_O_3(3D)_ RPC. Si element was precipitated on 5083, and O element formed a full and uniform oxide film on the Al_2_O_3(3D)_/5083 surface [32].

**Figure 22:**
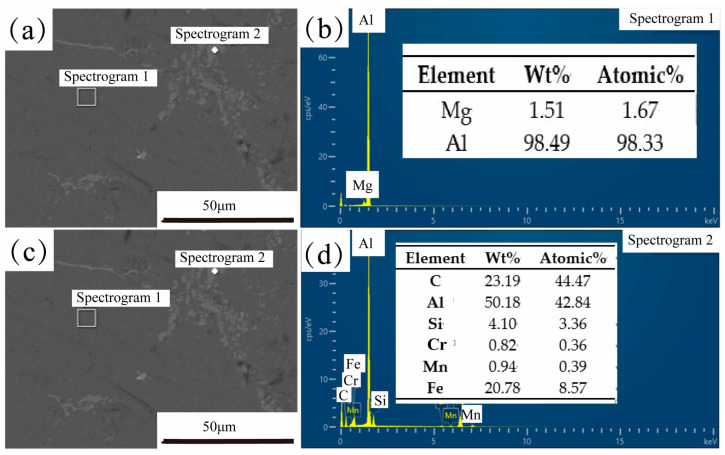
SEM image and EDS results of Al_2_O_3(3D)_/5083 from Al_2_O_3(3D)_ porosity of 15 PPI with liquid 5083Al at PV 0.4 m/s and PT 800 °C; (**a**) SEM image; (**b**) EDS of spectrogram l; (**c**) SEM image; (**d**) EDS of spectrogram 2.

Figure 22 shows SEM images and EDS results of Al_2_O_3(3D)_/5083 from Al_2_O_3(3D)_ porosity of 15 PPI with liquid 5083Al at PV 0.4 m/s and PT 800 °C. Point 1 contains 98.49% Al and 1.51% Mg, indicating that the material tested is an Al-Mg. Point 2, 50.18% Al, 23.19% C, 20.78% Fe, 4.1% Si, 0.82% Cu, and 0.94% Mn, indicating the presence of precipitates mainly containing Fe [33]. Infiltration kinetics was better in the case of the RMP route with liquid 5083Al with PV 0.4 m/s at PT 800 °C. The reactive infiltration was carried out at PV 0.4 m/s to prepare IPCs by reactive infiltration of liquid 5083Al into Al_2_O_3(3D)_ at 800 °C. The free surface tracking and the solidification phenomena for the infiltration of open-porous preforms was studied using both numerical simulation and experimental methods. The results provided insights into the optimal parameters for successful infiltration [34]. in this study may provide essential implication for the simulation and optimization of processing parameters in various infiltration casting systems.

## 5. Conclusions

In this investigation, in order to gain a deeper understanding of the infiltration and solidification processes of liquid 5083Al alloy into Al_2_O_3(3D)_ RPC during low-pressure infiltration process (LPIP), Al_2_O_3(3D)_ RPC preforms were simplified as Kelvin cells, and the infiltration and solidification processes of liquid 5083Al with pouring velocities (PV) of 0.4 m/s infiltrating into Al_2_O_3(3D)_ RPC preforms with varying porosities at different pouring temperatures (PT) were simulated using ProCAST software. The conclusions are the following:

1. During the infiltration process, the smaller the pore size of Al_2_O_3(3D)_ preform would cause the more work of resistance, the more loss of the energy of liquid 5083Al and the smaller PV.

2. The porosity of 65%~80% obtained better infiltration effect, indicating infiltration rate could be improved with reasonable porosity, leading to better preparation of Al_2_O_3(3D)_/5083 IPCs. 

3. The PV and the PT of the liquid 5083Al alloy was too low, and the liquid metal cannot fully penetrate into the Al_2_O_3(3D)_ RPC. Shrinkage pore distribution was more uniform, and the probability of shrinkage pore occurrence was small. The shrinkage porosity distribution is low and concentrated in Al_2_O_3(3D)_ RPC center.

4. Process parameters were optimized to obtain the proper PT of 800 °C and PV of 0.4 m/s, a composited casting has been infiltrated completely without any defects, such as not full filling, porosity or shrinkage. The predicted results show good agreement with the experimental data. It can be a useful method to the preparation of other metal matrix composites reinforced by RPC using infiltration casting.

## Figures and Tables

**Table 1:** Chemical composition of 5083 aluminum alloy.

Elements	Si	Cu	Mg	Zn	Mn	Ti	Cr	Fe	Al
Wt.%	0.4	0.03	4.5	0.27	0.50	0.15	0.07	0.15	Balance

**Table 2:** Boundary conditions of Al_2_O_3(3D)_/5083 IPCs simulated model during LPIP.

Volumes	Initial Temperature/°C	Boundary	HTC/ (W·m^−2^·°C^−1^)
graphite inlet graphite gate graphite mold liquid 5083Al Al_2_O_3(3D)_ RPC	250 250 250 740, 760, 800 540	graphite inlet & graphite gate liquid 5083Al & graphite mould liquid 5083Al & graphite inlet and gate liquid 5083Al & Al_2_O_3(3D)_ RPC	EQUIV 1416 1000 480

## Data Availability

Data sharing is not applicable for this article.

## References

[1] Kota N., Charan M.S., Laha T., Roy S. (2022). Review on development of metal/ceramic interpenetrating phase composites and critical analysis of their properties. Ceram. Int..

[2] Jiang L., Jiang Y., Yu L., Yang H., Li Z., Ding Y. (2019). Thermo-Mechanical Coupling Analyses for Al Alloy Brake Discs with Al_2_O_3_-SiC_(3D)_/Al Alloy Composite Wear-Resisting Surface Layer for High-Speed Trains. Materials.

[3] Yu L., Hao S., Nong X., Cao X., Zhang C., Liu Y., Yan Y., Jiang Y. (2021). Comparative Study on the Corrosion Resistance of 6061Al and SiC(3D)/6061Al Composite in a Chloride Environment. Materials.

[4] Schukraft J., Horny D., Schulz K., Weidenmann,K (2022). A. 3D modeling and experimental investigation on the damage behavior of an interpenetrating metal ceramic composite (IMCC) under compression. Mater Sci. Eng. A..

[5] Zhao D., Haijun S., Liu Y., Shen Z., Liu H., Guo Y., Li X., Dong D., Jiang H., Liu C. (2022). Ultrahigh-Strength Porous Ceramic Composites via a Simple Directional Solidification Process. Nano Lett..

[6] Etemadi R., Wang B., Pillai K.M., Niroumand B., Omrani E., Rohatgi P. (2018). Pressure infiltration processes to synthesize metal matrix composites—A review of metal matrix composites, the technology and process simulation. Mater. Manuf. Process..

[7] da Silva C.C., Volpato G.M., Fredel M.C., Tetzlaff U. (2019). Low-pressure processing and microstructural evaluation of unidirectional carbon fiber-reinforced aluminum-nickel matrix composites. J. Mater. Process.Tech..

[8] Akbarnejad S., Tilliander A., Sheng D.-Y., Jönsson P.G. (2022). Effect of Batch Dissimilarity on Permeability of Stacked Ceramic Foam Filters and Incompressible Fluid Flow: Experimental and Numerical Investigation. Metals.

[9] Du J., Chong X., Jiang Y., Feng J. (2015). Numerical simulation of mold filling process for high chromium cast iron matrix composite reinforced by ZTA ceramic particles. Int. J. Heat Mass Trans..

[10] Chang C.-Y. (2006). Numerical simulation of the pressure infiltration of fibrous preforms during MMC processing. Adv. Compos. Mater..

[11] Chang C.-Y. (2009). Simulation of molten metal through a unidirectional fibrous preform during MMC processing. J. Mater. Process. Tech..

[12] Guan J.T., Qi L.H., Jian L.I.U., Zhou J.M., Wei X.L. (2013). Threshold pressure and infiltration behavior of liquid metal into fibrous preform. Trans. Nonferrous Met. Soc. China.

[13] Regulski W., Szumbarski J., Łaniewski-Wołłk Ł., Gumowski K., Skibiński J., Wichrowski M., Wejrzanowski T. (2015). Pressure drop in flow across ceramic foams—A numerical and experimental study. Chem. Eng. Sci..

[14] Zabaras N., Samanta D. (2004). A stabilized volume-averaging for flow in porous media and binary alloy solidification processes. Int. J. Numer. Meth. Eng..

[15] Wehinger G.D., Heitmann H., Kraume M. (2016). An artificial structure modeler for 3D CFD simulations of catalytic foams. Chem. Eng. J..

[16] Nie Z., Lin Y., Tong Q. (2017). Numerical investigation of pressure drop and heat transfer through open cell foams with 3D Laguerre-Voronoi model. Int. J. Heat Mass. Trans..

[17] Buonomo B., di Pasqua A., Manca O., Nappo S., Nardini S. (2022). Entropy generation analysis of laminar forced convection with nanofluids at pore length scale in porous structures with Kelvin cells. Int. Commun. Heat Mass. Trans..

[18] Li Y., Yang B., Zhang M., Wang H., Gong W., Lai R., Li Y., Teng J. (2023). The corrosion behavior and mechanical properties of 5083Al-Mg alloy manufactured by additive friction stir deposition. Corros. Sci..

[19] Nkoua C., Josse C., Proietti A., Basseguy R., Blanc C. (2023). Corrosion behaviour of the microbially modified surface of 5083Aluminium alloy. Corros.Sci..

[20] Yu L., Zhang C., Liu Y., Yan Y., Xu P., Jiang Y., Cao X. (2022). Comparing the Corrosion Resistance of 5083Al and Al_2_O_3_3D/5083Al Composite in a Chloride Environment. Materials.

[21] Buonomo B., Pasqua A.D., Manca O., Sekrani G., Poncet S. (2020). Numerical Analysis on Pressure Drop and Heat Transfer in Nanofluids at Pore Length Scale in Open Metal Porous Structures with Kelvin Cells. Heat Trans. Eng..

[22] Dong C. (2016). Numerical Simulation of Metal Melt Flows in Mold Cavity with Ceramic Porous Media. Ceram-Silik..

[23] Lu S.-L., Xiao F.-R., Zhang S.-J., Mao Y.-W., Liao B. (2014). Simulation study on the centrifugal casting wet-type cylinder liner based on ProCAST. Appl. Therm. Eng..

[24] Liu L.-B., Hu C.-H., Zhang Y.-H., Song C.-J., Zhai Q.-J. (2023). Melt flow, solidification structures, and defects in 316 L steel strips produced by vertical centrifugal casting. Adv.Manuf..

[25] Hammel E.C., Ighodaro O.L.R., Okoli O.I. (2014). Processing and properties of advanced porous ceramics: An application based review. Ceram. Int..

[26] Zhang S., Zhu M., Zhao X., Xiong D., Wan H., Bai S., Wang X. (2016). A pore-scale, two-phase numerical model for describing the infiltration behaviour of SiC p /Al composites. Compos. Part A Appl. Sci. Manuf..

[27] Prakash S.A., Hariharan C., Arivazhagan R., Sheeja R., Raj V.A.A., Velraj R. (2021). Review on numerical algorithms for melting and solidification studies and their implementation in general purpose computational fluid dynamic software. J. Energy Storage.

[28] Kaur I., Singh P. (2021). Numerical investigation on conjugate heat transfer in octet-shape-based single unit cell thick metal foam. Int. Commun. Heat Mass Transf..

[29] Guo X., Liu R., Wang J., Shuai S., Xiong D., Bai S., Zhang N., Gong X., Wang X. (2021). 3D actual microstructure-based modeling of non-isothermal infiltration behavior and void formation in liquid composite molding. Appl. Math. Model..

[30] Lacoste E., Arvieu C., Mantaux O. (2018). Numerical Modeling of Fiber-Reinforced Metal Matrix Composite Processing by the Liquid Route: Literature Contribution. Metall. Mater. Trans. B..

[31] Nong X.D., Jiang Y.L., Fang M., Yu L., Liu C.Y. (2017). Numerical analysis of novel SiC_3D_/Al alloy co-continuous composites ventilated brake disc. Int. J. Heat Mass.Trans..

[32] Xue L., Wang F., Ma Z., Wang Y. (2015). Effects of surface-oxidation modification and heat treatment on silicon carbide 3D/AlCu 5 MgTi composites during vacuum-pressure infiltration. Appl. Surf. Sci..

[33] Ma X., Zhao Y.F., Tian W.J., Qian Z., Chen H.W., Wu Y.Y., Liu X.F. (2016). A novel Al matrix composite reinforced by nano-AlN(p) network. Sci. Rep..

[34] Potoczek M., Śliwa R. (2011). Microstructure and Physical Properties of AlMg/Al_2_O_3_ Interpenetrating Composites Fabricated by Metal Infiltration into Ceramic Foams. Arch. Metall. Mater..

